# Perceived Acceleration in Working Life and Meaning in Life: The Role of Sense of Agency and Urban–Rural Differences

**DOI:** 10.3390/bs16071226

**Published:** 2026-07-19

**Authors:** Yuqing Cui, Hui Lu, Siwen Song, Junxiu Wang

**Affiliations:** 1 School of Mental Health, Wenzhou Medical University, Wenzhou 325035, China; yuqing_choi@wmu.edu.cn (Y.C.); luhui@wmu.edu.cn (H.L.); 1548085450@wmu.edu.cn (S.S.); 2 Chinese Academy of Social Sciences, Beijing 100732, China

**Keywords:** technological acceleration, acceleration of social change, meaning in life, sense of agency, urban–rural differences

## Abstract

Drawing on social acceleration theory, this study examines how two perceived dimensions of acceleration in working life—technological acceleration and acceleration of social change—are associated with sense of agency and meaning in life among employed young adults in contemporary China. These two dimensions capture perceived increases in communication and information flow, as well as changing knowledge and skill demands in working life. Empirical evidence remains limited on how these perceived acceleration experiences are associated with agency and meaning in life, particularly across urban and rural contexts. Using data from the 2024–2025 Chinese Social Mentality Survey, we analyzed 5178 employed adults (aged 18–45). Structural equation modeling and multi-group analyses were used to examine the associations among technological acceleration, acceleration of social change, sense of agency, and meaning in life. The results showed that both measured acceleration dimensions were positively associated with presence of meaning and search for meaning, and these associations were partially statistically mediated by sense of agency. Notably, acceleration of social change shows stronger associations with sense of agency and search for meaning among rural participants, whereas sense of agency is more strongly associated with presence of meaning among urban participants. These findings suggest that the two measured dimensions of perceived acceleration in working life are not necessarily associated with lower meaning in life among employed young adults. Instead, they may be linked to meaning in life partly through sense of agency, highlighting the importance of considering urban–rural contexts.

## 1. Introduction

During the transition into employment, meaning in life often becomes unstable for young adults. This stage can be a critical window because several life tracks shift at once. Young people must quickly integrate identity and set goals across school-to-work moves, career development, and changing family roles (Hatano et al., 2024, 2025). At this time, job competition is intensifying, career paths feel less predictable, and social comparison is more visible (Hatano et al., 2024). As a result, many young adults increasingly doubt the association between effort and reward, and they experience instability in their sense of life direction (Kalleberg, 2009). Meaning in life is a core construct in existential psychology and refers to people’s subjective sense that life is understandable, purposeful, and significant (Steger, 2022). It is usually described with two related dimensions: presence of meaning and search for meaning. Presence of meaning captures whether life feels meaningful right now, while search for meaning captures ongoing exploration and meaning-making (Al-Mahrouqi et al., 2024; Uzun & Arslan, 2025). In contemporary digital society, young adults are increasingly immersed in algorithmic information environments, skill-updating pressure, and platform-based work rhythms. This makes it important to ask how shifting social time structures may be associated with meaning in life for young workers.

The concept of an acceleration society offers a useful lens for understanding growing time pressures in contemporary social life. Social acceleration is treated as a defining feature of late modernity rather than a set of isolated changes, and is commonly conceptualized as comprising three interrelated dimensions: technological acceleration, acceleration of social change, and acceleration of the pace of life (Hollstein & Rosa, 2023). These dimensions reinforce one another through an acceleration cycle, creating a self-propelling dynamic that intensifies temporal pressures at both societal and individual levels (Rosa, 2003). Technological acceleration refers to the increasing speed of goal-directed processes such as communication, production, and information processing (Ulferts et al., 2013). In contemporary working life, technological acceleration is reflected in faster communication, expanding information flows, shorter update cycles, and changing forms of digital coordination (Rosa & Smith, 2022). Acceleration of social change captures the faster turnover of social rules, skill demands, and value orientations (López-Deflory et al., 2023). This process produces the shrinking present, in which past experience becomes obsolete more quickly and future trajectories are harder to anticipate (Hsu & Elliott, 2015). In this study, the acceleration society serves as a macro-level background. Analytical attention is therefore placed on technological acceleration and acceleration of social change, the two dimensions most closely tied to contemporary employment experiences and most amenable to survey measurement. This focus is consistent with prior empirical research on acceleration in working life, particularly the General Acceleration Scale developed by Ulferts et al. (2013). In the context of employed young adults, technological acceleration is reflected in perceived increases in communication and information flow, whereas acceleration of social change is reflected in perceived increases in knowledge-updating and work-related skill demands. The third dimension—acceleration of the pace of life, typically associated with denser schedules and subjective time scarcity—is beyond the scope of the present analysis. Thus, the present study draws on social acceleration theory as a macro-level theoretical framework, but it does not operationalize social acceleration as a whole. Empirically, it examines two perceived dimensions of acceleration in working life—technological acceleration and acceleration of social change—because these were the dimensions measured in the survey.

Acceleration may relate to meaning in life in two opposing ways. One account predicts erosion of meaning under accelerating conditions. According to social acceleration theory, acceleration heightens obsolescence and everyday uncertainty (Hsu & Elliott, 2015). When experience becomes unstable, life is less likely to be perceived as coherent and understandable. Because perceived comprehensibility is a core foundation of meaning, its erosion undermines the presence of meaning (Lutz et al., 2023). From a broader social perspective, theories of liquid modernity suggest that increasingly fragile identities make it difficult to sustain coherent life narratives (Elliott, 2007). Network society dynamics may further amplify this process by intensifying constant connectivity and rapid information turnover (X. W. Chan et al., 2023). A competing account predicts meaning enhancement through empowerment and opportunity. Technological acceleration can be associated with higher efficiency and wider access to information, tools, and choices (Tarasiuk, 2024). Faster feedback and skill growth may be related to stronger goal pursuit and self-direction. Accelerating social change can open new routes and legitimize more diverse value commitments, which may be associated with stronger meaning search (Goyal & Iychettira, 2022). Evidence points in both directions, so the net association is not predetermined. Therefore, acceleration may either erode meaning or enhance it, depending on how it is experienced.

Sense of agency may be a mediator between acceleration and meaning in life. It refers to the subjective experience of being the author of one’s actions and believing that one’s behavior can shape outcomes in the external world (Zhang et al., 2022). Demand–control theory clarifies why this construct is especially consequential in fast-moving, high-demand contexts: when demands are high but control is low, strain becomes more likely, whereas greater control can shift the same demands toward challenge and effective engagement (Beehr et al., 2001). From a strain-based account, accelerating social change may be associated with reduced agency through repeated episodes of forced adaptation. Rules shift quickly, skills become outdated, and plans require constant revision, which can leave young workers feeling reactive rather than self-directed (Baquero et al., 2025). Technological acceleration may represent another layer, because always-on connectivity and continuous upskilling expectations can be associated with exhaustion and a sense of powerlessness (Singh et al., 2022). As agency declines, daily life may feel less structured and less predictable, and this may undermine a psychological basis of meaning in life (Martela & Ryan, 2016; Martela et al., 2018). A competing account suggests the opposite direction is also plausible. New technologies can expand action tools, improve planning and coordination, and provide clearer feedback, which may strengthen perceived control during goal pursuit. Rapid social change can also encourage proactive learning and deliberate re-planning, allowing individuals to regain control by adapting strategically. Evidence that self-regulatory capacity supports broad adjustment fits this agency-based view (Park et al., 2012). Therefore, sense of agency may offer a pathway through which acceleration may carry either negative or positive indirect effects on meaning in life, depending on how acceleration is experienced.

There may be urban–rural differences between these associations. China’s urban–rural divide is embedded in long-standing institutional structures. The household registration (hukou) system has historically constrained residential mobility and produced persistent inequalities in access to education, healthcare, employment, and public services between urban and rural areas (K. W. Chan & Buckingham, 2008). Even as urbanization has accelerated in recent decades, substantial disparities in labor-market conditions and opportunity structures remain (Fan, 2002). Rural employed young adults continue to face systematically different contexts from their urban counterparts in terms of the availability of stable employment, training resources, and social support networks (Nugin, 2020). Against this backdrop, differences in access to digital infrastructure and technologies may shape how technological acceleration is associated with perceived control, thereby altering its association with meaning in life (Bergdahl et al., 2023). Likewise, variations in labor-market structures across urban and rural areas may influence whether acceleration of social change is experienced as manageable or overwhelming, which could modify both its direct link to meaning and its indirect link through agency (Chen & Phanumartwiwath, 2025). Opportunity structures further condition these processes. In some contexts, acceleration may be more likely to undermine control, whereas in others it may facilitate agency and upward mobility (Bergdahl et al., 2023). Resource availability also matters, as the capacity to respond through training, job switching, or relocation may strengthen or weaken the connection between acceleration and agency, and between agency and meaning (Chen & Phanumartwiwath, 2025). From a theoretical perspective, uneven inclusion in information networks suggests that acceleration may be experienced with different intensity and consequences across contexts (Cui, 2025), while social acceleration theory emphasizes that acceleration is inherently comparative and reference-dependent (Van Dooremalen, 2021). Control-related resources are also unevenly distributed, and beliefs about control vary across social groups and institutional settings (Baquero et al., 2025). In sum, these considerations suggest that the strength and direction of the associations between acceleration, sense of agency, and meaning in life may differ between urban and rural employed young adults.

Despite strong theoretical foundations, prior research has rarely examined how technological acceleration and the acceleration of social change are associated with meaning in life. Moreover, little is known about the psychological factors that may statistically mediate these associations, and existing studies have largely overlooked whether these processes differ between urban and rural contexts. To address these gaps, the present study investigates how the two measured dimensions of perceived acceleration in working life—technological acceleration and acceleration of social change—are associated with meaning in life among employed young adults. It also tests the statistical mediating role of sense of agency and explores urban–rural differences in these associations. Accordingly, the following hypotheses and research question are proposed: (H1) Technological acceleration will be positively associated with sense of agency, presence of meaning, and search for meaning; sense of agency will statistically mediate the positive associations between technological acceleration and both dimensions of meaning in life. (H2) Acceleration of social change will be positively associated with sense of agency, presence of meaning, and search for meaning; sense of agency will statistically mediate the positive associations between acceleration of social change and both dimensions of meaning in life. Because existing research does not provide a sufficiently strong basis for specifying the exact direction of urban–rural differences in these structural associations, urban–rural variation is examined as a research question rather than as a directional hypothesis. (RQ1) How do the structural associations among technological acceleration, acceleration of social change, sense of agency, presence of meaning, and search for meaning differ between urban and rural employed young adults?

## 2. Materials and Methods

### 2.1. Participants and Data Collection

The data for this study were drawn from the Chinese Social Mentality Survey (CSMS), a nationwide survey conducted by the Research Center of Social Psychology, Chinese Academy of Social Sciences. The CSMS is a cross-sectional survey carried out between December 2024 and February 2025 using stratified, probability proportional to size sampling based on the Seventh National Population Census. Covering 30 provinces, autonomous regions, and municipalities, the survey provides broad national representativeness and includes topics such as social needs, social values, social mentality, AI-related issues, and demographic information.

Participants were recruited through a professional survey organization and completed the questionnaire during face-to-face interviews. Prior to data collection, all the participants were informed of the survey’s purpose, their right to withdraw at any time, and that no personally identifiable information would be recorded or disclosed. Participation was voluntary and implied consent to include responses in the research. A total of 10,294 individuals aged 18 to 70 years completed the survey.

The present study focused on employed young adults. To define the target population, an age range of 18 to 45 years was applied, which captures the developmental period from young adulthood to early midlife. This life stage is characterized by career establishment and family formation and is considered particularly sensitive to perceived social acceleration and related stressors. In addition, employment status was used as a further inclusion criterion. Participants who reported being currently employed, engaged in non-standard employment (e.g., freelance or gig work), or combining agricultural work with off-farm employment were included to reflect diverse employment arrangements. After applying these selection criteria, the final analytic sample consisted of 5178 participants. Given the study’s focus on employed young adults, a quantitative design was used to examine theoretically specified associations in a large-scale survey sample and to compare structural paths across urban and rural groups. Urban–rural residence was treated as the grouping variable in the multi-group analysis rather than as an analytic inclusion criterion.

### 2.2. Measures

#### 2.2.1. Background Factors

Background factors were collected on community type (rural or city area), age, sex, educational level, personal monthly income, and socioeconomic status (SES).

#### 2.2.2. Meaning in Life

Meaning in life was assessed using the Chinese version of the Meaning in Life Questionnaire (MLQ-C; Wang, 2013). The scale consists of 10 items and comprises two subscales: presence of meaning and search for meaning. The presence of meaning subscale assesses the extent to which individuals perceive their lives as meaningful, with a sample item being “I understand the meaning of my life.” The search for meaning subscale measures individuals’ motivation to seek meaning in life, with a sample item being “I am searching for something that makes my life feel meaningful.” All items are rated on a 7-point Likert scale (1 = absolutely untrue to 7 = absolutely true), with higher scores indicating higher levels of the corresponding construct. In the present study, Cronbach’s α was 0.71 and 0.76 for the presence of meaning subscale and search for meaning subscale.

#### 2.2.3. Sense of Agency

Sense of agency was measured by using an adapted Chinese version of the Sense of Agency Scale (Zhang et al., 2022). It consisted of four items assessing subjective experiences of agency. Sample items included “I feel that I am fully in control of my actions” and “My behavior is planned by me from beginning to end.” The participants rated each item on a 7-point Likert scale (1 = strongly disagree to 7 = strongly agree), with higher scores indicating a stronger sense of agency. In the present study, the Cronbach’s α was 0.89.

#### 2.2.4. Technological Acceleration

Technological acceleration was assessed using the adapted technological acceleration subscale of the General Acceleration Scale (Ulferts et al., 2013). It consisted of two items, which asked participants to compare their current life situation with that of two years ago and rate the changes in the following areas: the amount of information received and the number of emails or messages received daily (e.g., through email or WeChat). It should be noted that these items primarily reflect perceived increases in information and communication volume in working life. They do not directly measure automated technology adoption, or all aspects of technological acceleration. Therefore, findings related to this dimension should be interpreted mainly as evidence concerning perceived information- and communication-related acceleration. The participants rated these items on a 7-point Likert scale (1 = significantly decreased to 7 = significantly increased). Higher scores indicated a greater technological acceleration in their daily lives. In the present study, the Cronbach’s α was 0.73.

#### 2.2.5. Acceleration of Social Change

Acceleration of social change was assessed using the social change acceleration subscale of the General Acceleration Scale (Ulferts et al., 2013). The subscale consisted of two items assessing perceived changes in social and work-related demands. The participants were asked to compare their current life situation with that of two years ago and rate changes in the following areas: the additional skills required (e.g., conflict resolution, problem-solving, and teamwork skills) and the frequency of updating knowledge required for work. These items capture a specific facet of acceleration of social change in working life, namely perceived increases in skill and knowledge-updating demands. Thus, this measure should be interpreted as an abbreviated indicator of acceleration of social change rather than as a comprehensive assessment of the full theoretical construct. All items were rated on a 7-point Likert scale (1 = significantly decreased to 7 = significantly increased). Higher scores indicated a greater perceived acceleration of social change. In the present study, the Cronbach’s α was 0.75.

### 2.3. Statistical Analysis

Descriptive statistics were conducted for all study variables. Univariate linear regression analyses examined associations between background factors and outcome variables, including presence of meaning and search for meaning. Pearson correlation coefficients assessed relationships among continuous variables. Structural equation modeling (SEM) was conducted to test whether sense of agency mediated associations between technological acceleration/acceleration of social change and outcome variables. A consistent set of theoretically relevant background variables was included as covariates in the SEM analyses, including sex, age, educational level, personal monthly income, socioeconomic status, and community type where appropriate. Before conducting multi-group SEM, measurement invariance across urban and rural participants was tested for the main latent constructs, including the two acceleration dimensions in working life, sense of agency, and meaning in life. Configural, metric, and scalar invariance models were estimated sequentially. Model comparisons were evaluated using changes in fit indices, with ΔCFI ≤ 0.010, ΔRMSEA ≤ 0.015, and ΔSRMR ≤ 0.030 for metric invariance and ΔSRMR ≤ 0.010 for scalar invariance indicating acceptable invariance (Cheung & Rensvold, 2002). Multi-group SEM was performed to examine potential urban–rural differences in the mediation model. Model parameters were compared across the urban and rural groups. Satisfactory model fit indices were evaluated using the Comparative Fit Index (CFI) ≥ 0.90, Tucker–Lewis Index (TLI) ≥ 0.90, Root Mean Square Error of Approximation (RMSEA) ≤ 0.08, and Standardized Root Mean Square Residual (SRMR) ≤ 0.08 (Kline, 2016). SEM analyses were conducted by using Mplus 8.3 while the other analyses were performed by SPSS 24.0. Statistical significance was defined as a two-tailed *p* < 0.05. Indirect effects were estimated using the MODEL INDIRECT command in Mplus 8.3 with 5000 bootstrap resamples. The statistical significance of mediation was evaluated using 95% bootstrapped confidence intervals, with indirect effects considered significant when the confidence interval did not include zero.

## 3. Results

### 3.1. Descriptive Analyses

As presented in Table 1, the mean age of participants was 33.64 years (SD = 6.59). Most participants resided in urban areas (69.9%), and females accounted for 49.9% of the sample. A majority of the participants had attained a college degree or higher (71.1%). Most participants reported a monthly personal income ranging from 4001 to 8000 yuan. The mean socioeconomic status score was 5.21 (SD = 1.35).

Regarding key study variables, the mean scores (SD) for technological acceleration, acceleration of social change, sense of agency, presence of meaning, and search for meaning were 9.04 (2.04), 9.10 (1.82), 19.53 (4.75), 24.13 (4.20), and 23.82 (4.27), respectively.

### 3.2. The Associations Between Background Factors and Outcome Variables

Univariate regression analyses showed that participants living in urban areas reported lower levels of presence of meaning (*β* = −0.05). Male participants were also less likely to report presence of meaning (*β* = −0.06), whereas having a college education or higher was positively associated with presence of meaning (*β* = 0.07). Compared with individuals earning 4000 yuan or less per month, those with monthly incomes above 8000 yuan reported higher presence of meaning (*β* = 0.15). In addition, age (*β* = 0.14) and socioeconomic status (*β* = 0.15) were positively associated with presence of meaning.

Regarding search for meaning, urban residence was associated with lower levels of search for meaning (*β* = −0.12). Compared with the lowest income group, participants earning 4001–8000 yuan (*β* = −0.08) and above 8000 yuan (*β* = −0.18) were less likely to engage in search for meaning. Socioeconomic status was positively associated with search for meaning (*β* = 0.15). In contrast, sex, educational level, and age were not significantly associated with search for meaning. These results are presented in Table 2.

### 3.3. Pearson Correlations

As shown in Table 3, technological acceleration, acceleration of social change, sense of agency, presence of meaning, and search for meaning were positively correlated with each other (*r* ranges from 0.11 to 0.44).

### 3.4. SEM

**Figure 1:**
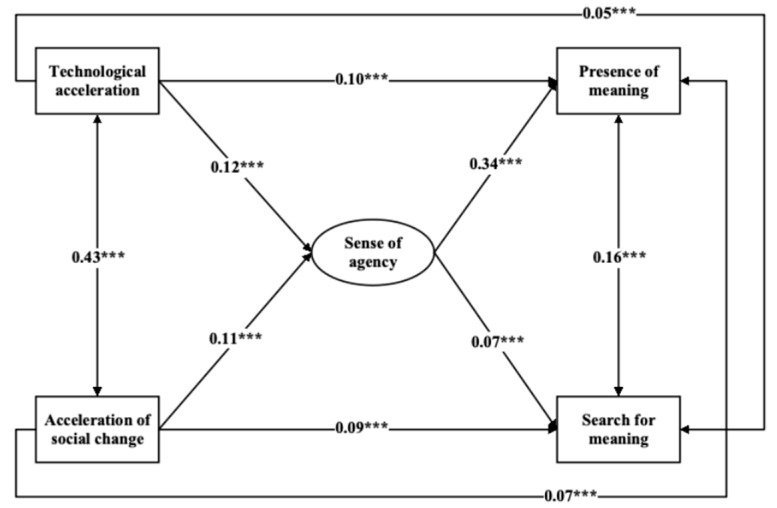
Structural equation modeling. The standardized coefficients are reported. The models were adjusted for background factors, including community type, sex, educational level, personal monthly income, age, and social economic status. *** *p* < 0.001.

Figure 1 presents the results of the structural equation model. The model demonstrated a satisfactory fit to the data (*CFI* = 0.962, *TLI* = 0.938, *RMSEA* = 0.048, *SRMR* = 0.031). Technological acceleration was positively associated with sense of agency (*β* = 0.12, *p* < 0.001), which was, in turn, positively associated with presence of meaning (*β* = 0.34, *p* < 0.001). In addition, technological acceleration showed a significant direct association with presence of meaning (*β* = 0.10, *p* < 0.001), indicating a partial mediating role of sense of agency. The bootstrapped indirect effect of technological acceleration on presence of meaning through sense of agency was significant (*β* = 0.031, SE = 0.005, 95% CI [0.020, 0.041], *p* < 0.001). A similar pattern was observed for search for meaning. Technological acceleration was positively associated with sense of agency (*β* = 0.12, *p* < 0.001), which was positively associated with search for meaning (*β* = 0.07, *p* < 0.001). Technological acceleration also had a significant direct association with search for meaning (*β* = 0.05, *p* < 0.001) and the indirect effect through sense of agency was also significant (*β* = 0.006, SE = 0.001, 95% CI [0.003, 0.009], *p* < 0.001), suggesting partial mediation.

Acceleration of social change was also positively associated with sense of agency (*β* = 0.11, *p* < 0.001). Sense of agency, in turn, was positively associated with presence of meaning (*β* = 0.34, *p* < 0.001). Acceleration of social change retained a significant direct association with presence of meaning (*β* = 0.07, *p* < 0.001), supporting partial mediation. The bootstrapped indirect effect of acceleration of social change on presence of meaning through sense of agency was significant (*β* = 0.038, SE = 0.005, 95% CI [0.028, 0.049], *p* < 0.001). Likewise, sense of agency was positively associated with search for meaning (*β* = 0.07, *p* < 0.001), and acceleration of social change showed a significant direct association with search for meaning (*β* = 0.09, *p* < 0.001), with a significant indirect effect through sense of agency (*β* = 0.008, SE = 0.001, 95% CI [0.004, 0.011], *p* < 0.001). The standardized indirect effects are presented in Supplementary Table S1.

### 3.5. Measurement Invariance Across Urban and Rural Groups

As shown in Supplementary Table S2, all configural models showed acceptable fit. The metric and scalar models did not show meaningful deterioration in model fit compared with the preceding models, with changes in CFI, RMSEA, and SRMR remaining within the recommended thresholds. These findings supported scalar invariance for the two acceleration dimensions in working life, sense of agency, and meaning in life across the urban and rural groups, indicating that subsequent comparisons of structural paths across the groups were appropriate.

### 3.6. Multi-Group SEM

A multi-group SEM was conducted to examine whether structural paths differed between urban and rural participants. The unconstrained model, in which all structural paths were freely estimated across the groups, demonstrated an acceptable fit to the data (*χ*^2^(18) = 175.87, *CFI* = 0.918, *SRMR* = 0.037, *RMSEA* = 0.060). In contrast, the constrained model, which imposed equality constraints on all structural paths across the groups, showed a significantly poorer fit (*χ*^2^(26) = 221.49, *CFI* = 0.898, *SRMR* = 0.044, *RMSEA* = 0.055). Model comparisons indicated a meaningful decline in fit (Δ*χ*^2^(8) = 45.62, *p* < 0.001), suggesting that structural paths differed between urban and rural participants.

Further examination of individual path constraints revealed several significant urban–rural differences (Table 4). The path from acceleration of social change to search for meaning varied significantly across the groups (Wald test estimate = 7.50, *p* = 0.006). Among rural participants, acceleration of social change was positively associated with search for meaning (*β* = 0.13, *p* < 0.001), whereas this association was weaker, though still significant, among urban participants (*β* = 0.04, *p* = 0.013). A similar group difference was observed for the path from acceleration of social change to sense of agency (Wald test estimate = 6.50, *p* = 0.011). Specifically, acceleration of social change showed a stronger positive association with sense of agency in rural participants (*β* = 0.18, *p* < 0.001) than in urban participants (*β* = 0.09, *p* < 0.001).

In addition, the association between sense of agency and presence of meaning differed significantly between the groups (Wald test estimate = 5.21, *p* = 0.023). Sense of agency was positively associated with presence of meaning in both rural (*β* = 0.31, *p* < 0.001) and urban participants (*β* = 0.37, *p* < 0.001), with a stronger association observed in the urban group. Finally, a significant urban–rural difference was found for the path from sense of agency to search for meaning (Wald test estimate = 9.42, *p* = 0.002). This association was stronger among rural participants (*β* = 0.17, *p* < 0.001) than among urban participants (*β* = 0.07, *p* < 0.001). Detailed path information is also presented in Supplementary Figures S1 and S2.

## 4. Discussion

This study draws on social acceleration theory as a macro-level framework but empirically focuses on two measured dimensions of perceived acceleration in working life: technological acceleration and acceleration of social change. These findings therefore should not be interpreted as evidence about social acceleration as a whole. Rather, they concern how these two measured acceleration dimensions are associated with meaning in life among employed young adults. It further tests the mediating role of sense of agency and explores urban–rural differences in these relationships. The results show that both forms of acceleration are positively associated with sense of agency and with both dimensions of meaning in life. Sense of agency partially mediated such associations. In addition, multi-group SEM reveals significant differences in several structural paths between urban and rural participants, indicating that the associations among acceleration, sense of agency, and meaning in life are context-sensitive. These findings suggest that, in the contemporary employment context marked by rapid digital transformation, technological acceleration and acceleration of social change are not inevitably associated with lower meaning in life among employed young adults. Instead, both dimensions were positively associated with presence of meaning and search for meaning, and these associations were partially linked to sense of agency. This pattern suggests that perceived acceleration may coexist with a stronger sense of personal agency and meaning-making, rather than only with psychological strain. Given the cross-sectional design, however, the directionality and causal nature of these associations cannot be established from the current data.

A key issue that requires deeper explanation is why, although social acceleration theory often emphasizes disorder and alienation, the two measured dimensions of perceived acceleration in working life showed positive associations with agency and meaning in the present study. One important reason is that the present study does not examine acceleration of the pace of life, which is typically associated with denser schedules and time scarcity. Instead, it focuses on two dimensions more directly linked to structural change in contemporary working life. For employed young adults, these forms of acceleration are more likely to be experienced as identifiable changes in external rules and usable improvements in tools, rather than as direct losses of time (Santarius & Bergener, 2020). In this sense, technological acceleration and acceleration of social change may not be experienced only as pressure. They may also be experienced as providing clearer feedback and more visible opportunities for adjustment (Geels & Ayoub, 2023). Rapid tool iteration may be associated with perceived improvements in task efficiency, information access, and coordination, while frequent shifts in rules and skill demands, although uncertain, may make expectations about learning and competitiveness more concrete and actionable (Tang et al., 2023). During the employment stage, goal orientation and skill accumulation are central developmental tasks. When change is perceived as a demand that can be responded to, it is more likely to trigger active learning and self-regulation rather than lead directly to a collapse of meaning (Hirschi & Koen, 2021). This resource-oriented interpretation should be treated cautiously. In the present study, the two measured dimensions of acceleration in working life—technological acceleration and acceleration of social change—were positively associated with sense of agency, presence of meaning, and search for meaning. This pattern suggests that some forms of perceived acceleration may coexist with agency and meaning-making. However, this interpretation should not be generalized to acceleration of the pace of life, to other forms of acceleration not measured here, or to populations with fewer adaptive resources.

The results provide statistical support for the proposed mediation hypotheses (H1 and H2), indicating that sense of agency was associated with both dimensions of acceleration and with meaning in life. In contexts characterized by accelerating change, individuals who perceive themselves as capable of initiating action and influencing outcomes may be more likely to interpret changing demands as manageable and to integrate them into personal planning (Frazier et al., 2021). In this sense, sense of agency may help explain the indirect associations between acceleration in working life and meaning in life. The significant indirect associations observed in the model suggest that sense of agency accounts for part of the statistical association between acceleration and subjective meaning. However, given the cross-sectional design, these indirect associations should not be interpreted as evidence of a causal mechanism.

It is also noteworthy that presence of meaning and search for meaning were positively correlated in the present sample (r = 0.25), and both were positively associated with the two acceleration dimensions. This pattern deserves attention because prior research has often suggested that presence of meaning and search for meaning may be negatively associated, particularly when search for meaning reflects a perceived lack of meaning (Steger et al., 2008). However, the relationship between the two dimensions is not necessarily uniform across populations and developmental contexts. In the present sample of employed young adults, search for meaning may not simply indicate a deficit in meaning presence. Rather, it may reflect an active process of goal clarification, self-development, and future-oriented adjustment during career formation. In this context, individuals may perceive their current lives as meaningful while also continuing to search for new goals, directions, and possibilities. This interpretation is consistent with studies suggesting that the association between presence of meaning and search for meaning can vary across age groups, developmental stages, and cultural contexts (Li et al., 2021).

Within this mediated framework, an important structural pattern emerges: sense of agency is much more strongly associated with presence of meaning than with search for meaning (0.34 vs. 0.07). This difference reflects the distinct psychological functions of the two meaning dimensions. Presence of meaning primarily concerns whether life feels coherent, understandable, and purposeful, all of which depend heavily on a stable sense of order and control (Subaşı, 2024). Higher sense of agency allows individuals to organize daily activities, anticipate outcomes, and maintain continuity in life narratives, thereby directly strengthening presence of meaning (Wenning, 2021). By contrast, search for meaning reflects an ongoing process of exploration and identity negotiation (Hatano et al., 2022). Although sense of agency can facilitate exploration by providing confidence and motivation, meaning search is also driven by exposure to new possibilities and unresolved questions, and thus depends less exclusively on perceived control (Seubert et al., 2024). As a result, agency plays a supportive but less central role in meaning search than in meaning presence. This asymmetry helps explain why the mediation through sense of agency is stronger for presence of meaning than for search for meaning.

The findings on urban–rural differences provide important boundary conditions and help explain why similar acceleration processes may yield different psychological outcomes across contexts. Multi-group SEM indicated that the associations from acceleration of social change to sense of agency and to search for meaning were stronger among rural participants. One possible interpretation is that, in the context of China’s persistent urban–rural divide, changes in social rules and skill demands may carry more distinctive practical meaning for rural employed young adults. Prior research has shown that rural–urban inequalities in China have long been shaped by institutional arrangements, labor-market segmentation, and unequal access to educational and economic resources (Wu & Treiman, 2004; K. W. Chan & Buckingham, 2008; Treiman, 2012). Although these structural constraints may limit opportunities, periods of rapid social and occupational change may also make skill acquisition, mobility, and adaptation more salient for rural employed young adults. In this sense, changing skill demands may be interpreted not only as pressure but also as potential signals of life-course adjustment and upward mobility. This may help explain why acceleration of social change was more strongly associated with sense of agency and search for meaning among rural participants. However, this explanation should be treated as a tentative theoretical interpretation rather than as a directly tested mechanism. The present survey did not measure specific structural factors such as migration costs, actual opportunity structures, labor-market constraints, or digital infrastructure quality. Future research incorporating these contextual measures is needed to evaluate whether and how these structural conditions explain urban–rural differences in the associations among acceleration, agency, and meaning in life. The cross-group pattern also suggests that sense of agency may be related to different dimensions of meaning in urban and rural contexts. In urban settings, sense of agency was more strongly associated with presence of meaning. One possible interpretation is that, in highly competitive and institutionally dense urban environments, agency may be closely related to the capacity to organize complexity, maintain control over work rhythms, and sustain coherent life plans. This interpretation is consistent with prior research suggesting that agency, control, and self-regulatory capacity are closely associated with coherence, goal pursuit, and meaning in life (Park, 2010; Martela et al., 2018). When individuals perceive themselves as able to manage competing demands and maintain direction, they may be more likely to report a stable sense of meaning in life. In rural contexts, by contrast, sense of agency was more strongly associated with search for meaning. One possible interpretation is that, for rural employed young adults facing changing skill demands and potential mobility opportunities, agency may be more closely associated with exploration, goal adjustment, and the search for new life directions. This interpretation is consistent with research emphasizing the role of agency and self-regulation in adaptive responses to changing life conditions (Park et al., 2012; Zhang et al., 2022). However, these interpretations should be treated as tentative rather than as directly tested mechanisms. The present survey did not directly measure contextual resources, mobility opportunities, or structural constraints; future research should incorporate such measures to examine why agency may relate to different dimensions of meaning across urban and rural contexts.

The findings extend existing theory in several ways. First, the findings complicate a uniformly negative interpretation of perceived acceleration in working life by showing that the two measured acceleration dimensions were positively associated with sense of agency and meaning in life in this sample. Second, by distinguishing presence of meaning and search for meaning, the study suggests that these two acceleration dimensions may be associated with different dimensions of meaning in life. Third, the urban–rural multi-group results highlight the context sensitivity of acceleration–agency–meaning associations, supporting the treatment of social structure as a boundary condition rather than merely a control factor.

The findings should also be considered in relation to cross-cultural specificity. The present study is based on a Chinese sample, and the observed positive associations between acceleration in working life, sense of agency, and meaning in life should not be interpreted as universal patterns. These associations may be particularly salient in a social context marked by rapid socioeconomic transformation, strong educational and occupational competition, and persistent urban–rural inequalities. In such a context, technological acceleration and acceleration of social change may be experienced not only as sources of pressure, but also as signals of adaptation, mobility, and self-development. However, these interpretations may not apply in the same way to societies with different welfare systems, labor-market structures, levels of economic development, or urbanization trajectories. The theoretical frameworks used in this study, including social acceleration theory and demand–control theory, provide useful analytical tools, but their application to the Chinese context also raises questions about cultural and institutional specificity. Cross-cultural replications are therefore needed before broader claims about the generalizability of these findings can be made.

Several limitations should be noted. First, the cross-sectional design precludes causal inference. Although the theoretical model was specified in terms of technological acceleration, acceleration of social change, sense of agency, and meaning in life, the observed associations cannot establish temporal ordering or causality. It is also possible that individuals with a stronger sense of agency or higher meaning in life perceive accelerating work and social changes more positively. Longitudinal or experimental designs are therefore needed to examine the directionality of these associations. Second, the use of self-report measures may introduce common method bias and measurement error; in particular, the presence of meaning subscale showed acceptable but relatively modest internal consistency (Cronbach’s α = 0.71). Third, this study did not include acceleration of the pace of life, the dimension of social acceleration most closely related to time scarcity and subjective overwhelm. Therefore, the findings should be interpreted as applying mainly to the two measured dimensions of acceleration in working life—technological acceleration and acceleration of social change—rather than to social acceleration as a whole. Finally, although urban–rural differences were identified, the structural sources of these differences, such as digital infrastructure, access to training, migration costs, labor-market opportunities, and social support networks, were not directly measured. Future research should use longitudinal or experimental designs; incorporate all three dimensions of social acceleration theory, including acceleration of the pace of life; and examine these associations across more diverse populations and institutional contexts.

## 5. Conclusions

The present study examined how two measured dimensions of perceived acceleration in working life—technological acceleration and acceleration of social change—were associated with meaning in life among employed young adults. It also tested the statistical mediating role of sense of agency and explored urban–rural differences in these associations. The findings suggest that the two measured dimensions of perceived acceleration in working life are not necessarily associated with lower meaning in life. Instead, they were positively associated with both presence of meaning and search for meaning, and these associations were partially linked to sense of agency. Multi-group analyses further indicated that several structural paths differed between urban and rural participants. Overall, these findings suggest that rapid changes in working life may coexist with agency and meaning-making among employed young adults, but such associations are shaped by social context. These findings highlight the importance of integrating macro-level time structures with concrete opportunity structures when examining how acceleration in working life is associated with agency and meaning in life. Future research should further examine whether these associations are observed in other cultural and institutional contexts.

## Figures and Tables

**Table 1:** Sample characteristics and descriptive statistics of the study variables.

**Categorical Variable**	***n***	**%**
Community type		
Rural	1559	30.1
City	3619	69.9
Sex		
Female	2584	49.9
Male	2594	50.1
Educational level		
High school or below	1494	28.9
College or above	3684	71.1
Personal monthly income		
4000 Yuan below	1290	24.9
4001–8000 Yuan	3134	60.5
Above 8000 Yuan	754	14.6
**Continuous Variable**	**Mean**	**SD**
Age	33.64	6.59
Social economic status	5.21	1.35
Technology acceleration	9.04	2.04
Acceleration of social change	9.10	1.82
Sense of agency	19.53	4.75
Presence of meaning	24.13	4.20
Search for meaning	23.82	4.27

**Table 2:** The associations between background variables and PM/SM.

Variable	Presence of Meaning		Search for Meaning	
	*β*	*p*	*β*	*p*
Community type				
Rural	Reference		Reference	
City	−0.05	<0.001	−0.12	<0.001
Sex				
Female	Reference		Reference	
Male	−0.06	<0.001	−0.01	0.641
Educational level				
High school or below	Reference		Reference	
College or above	0.07	<0.001	−0.02	0.131
Personal monthly income				
4000 Yuan or below	Reference		Reference	
4001–8000 Yuan	−0.01	0.776	−0.08	<0.001
Above 8000 Yuan	0.15	<0.001	−0.18	<0.001
Age	0.14	<0.001	−0.02	0.140
Social economic status	0.15	<0.001	0.07	<0.001

Reference = reference group.

**Table 3:** Pearson correlation matrix of the study variables.

Variable	1	2	3	4	5
Technology acceleration	1				
Acceleration of social change	0.44 ***	1			
Sense of agency	0.15 ***	0.16 ***	1		
Presence of meaning	0.18 ***	0.17 ***	0.40 ***	1	
Search for meaning	0.11 ***	0.15 ***	0.13 ***	0.25 ***	1

***, *p* < 0.001.

**Table 4:** Multi-group analysis across community type.

Path	Rural		City		Wald Test	*p*
	*β*	*p*	*β*	*p*	Estimate	
TA → PM	0.07	0.007	0.12	<0.001	3.70	0.054
ASC → SM	0.13	<0.001	0.04	0.013	7.50	0.006
TA → SM	0.06	0.021	0.05	0.010	0.04	0.847
ASC → PM	0.15	<0.001	0.11	<0.001	0.62	0.433
TA → SA	0.15	<0.001	0.09	<0.001	2.99	0.084
ASC → SA	0.18	<0.001	0.09	<0.001	6.50	0.011
SA → PM	0.31	<0.001	0.37	<0.001	5.21	0.023
SA → SM	0.17	<0.001	0.07	<0.001	9.42	0.002

TA = technology acceleration, ASC = acceleration of social change, PM = presence of meaning, SM = search for meaning, SA = sense of agency.

## Data Availability

The data analyzed in this study were obtained from the Chinese Social Mentality Survey (CSMS) conducted by the Chinese Academy of Social Sciences (CASS). Restrictions apply to the availability of these data, which were used under license/permission for the current study. The data are available from the original data provider upon reasonable request.

## Supplementary Materials

The following supporting information can be downloaded at https://www.mdpi.com/article/10.3390/bs16071226/s1, Figure S1: Structural equation model for rural participants; Figure S2: Structural equation model for urban participants; Table S1: Standardized indirect effects through sense of agency; Table S2: Measurement invariance across urban and rural groups.
