# Supplementary material for: Perceived Acceleration in Working Life and Meaning in Life: The Role of Sense of Agency and Urban–Rural Differences
Source: Behav Sci (Basel). 2026 Jul 19;16(7):1226. doi: 10.3390/bs16071226 (PMC13405711; doi:10.3390/bs16071226)
Supplement: Supplementary file 1 [file behavsci-16-01226-s001.zip › Supplementary Figures.pdf]

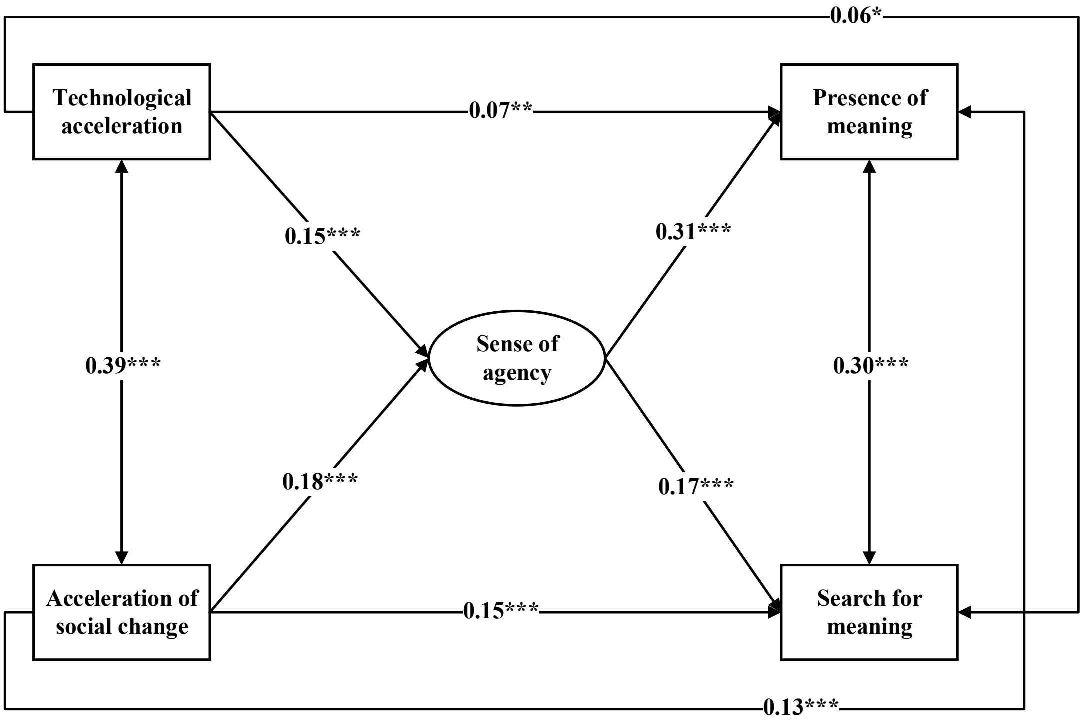

Supplementary Figure S1 Structural equation model for rural participants. *$p < .05$; **$p < .01$; ***$p < .001$.

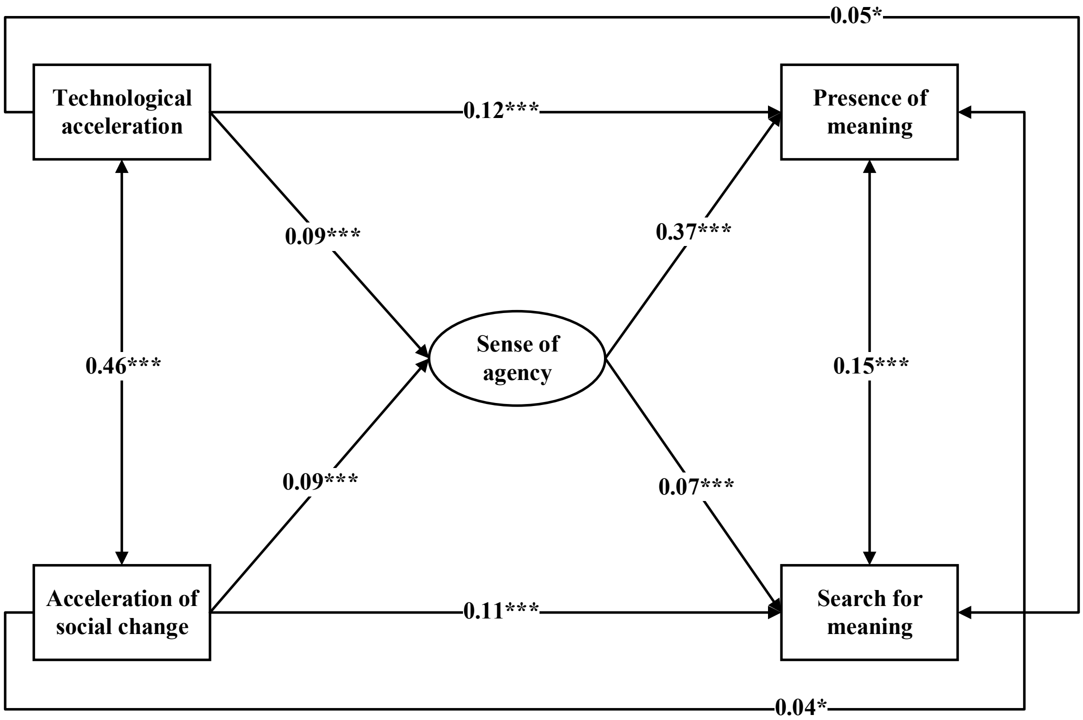

Supplementary Figure S2 Structural equation model for urban participants. *p < .05; **p < .01; ***p < .001.
