# Supplementary material for: Perceived Acceleration in Working Life and Meaning in Life: The Role of Sense of Agency and Urban–Rural Differences
Source: Behav Sci (Basel). 2026 Jul 19;16(7):1226. doi: 10.3390/bs16071226 (PMC13405711; doi:10.3390/bs16071226)
Supplement: Supplementary file 1 [file behavsci-16-01226-s001.zip › Supplementary Tables.pdf]

Supplementary Table S1 Standardized indirect effects through sense of agency

| Indirect effect | *β* | *SE* | 95% CI | *p* |
|---|---|---|---|---|
| TA → SA → PM | 0.031 | 0.005 | (0.020, 0.041) | < 0.001 |
| TA → SA → SM | 0.006 | 0.001 | (0.003, 0.009) | < 0.001 |
| ASC → SA → PM | 0.038 | 0.005 | (0.028, 0.049) | < 0.001 |
| ASC → SA → SM | 0.008 | 0.001 | (0.004, 0.011) | < 0.001 |

TA = technological acceleration; ASC = acceleration of social change; SA = sense of agency; PM = presence of meaning; SM = search for meaning. Standardized indirect effects are reported. Confidence intervals were estimated using bootstrapping with 5,000 resamples.

Supplementary Table S2 Measurement invariance across urban and rural groups

| **Variable** | **Model** | **CFI** | **TLI** | **RMSEA** | **SRMR** | **ΔCFI** | **ΔRMSEA** | **ΔSRMR** |
|---|---|---|---|---|---|---|---|---|
| Social acceleration | Configural Invariance | 0.950 | 0.943 | 0.038 | 0.033 | - | - | - |
| | Metric Invariance | 0.949 | 0.936 | 0.035 | 0.035 | 0.001 | 0.003 | 0.002 |
| | Scalar Invariance | 0.945 | 0.934 | 0.033 | 0.036 | 0.004 | 0.002 | 0.001 |
| Sense of agency | Configural Invariance | 0.937 | 0.928 | 0.028 | 0.037 | - | - | - |
| | Metric Invariance | 0.937 | 0.925 | 0.021 | 0.039 | 0.000 | 0.007 | 0.002 |
| | Scalar Invariance | 0.933 | 0.920 | 0.018 | 0.041 | 0.004 | 0.003 | 0.002 |
| Meaning in life | Configural Invariance | 0.953 | 0.938 | 0.055 | 0.051 | - | - | - |
| | Metric Invariance | 0.953 | 0.944 | 0.052 | 0.052 | 0.000 | 0.003 | 0.001 |
| | Scalar Invariance | 0.950 | 0.947 | 0.051 | 0.054 | 0.003 | 0.001 | 0.002 |
